# Supplementary material for: Chromothripsis during telomere crisis is independent of NHEJ, and consistent with a replicative origin
Source: Genome Res. 2019 May;29(5):737–49. doi: 10.1101/gr.240705.118 (PMC6499312; doi:10.1101/gr.240705.118)
Supplement: Supplemental Material [file supp_gr.240705.118_Supplemental_file_1.zip › contigs/annotated_contigs/DB48/contig.2.DB48_length_375_mean_cov_3.89066666667.docx]

**DB48_length_375_mean_cov_3.89066666667**

```
AA|AATAAAACAAAAAAAAAAAAAAAAAAAAAAAAAAAAAAAAAAAAAAAAAAAA|CAATATTAGGTATTCCAAAATA|AAAAAAAAAA
   >chr2:88206930-88206982 + E=8e-17 p=0e+00                                 >chr7:527
AAACAAAAAAATAAAACACACACAGTTGGAAAAACTGTGAATGATGTAAAGGAAACAATAGCTGTGAAAATAAATTTTAAAGGAATATA
89452-52789751 + E=8e-166
CTTTGATTAAGCTTTGAGAGATTATCACTTAAGTGGCACTTAAGTTTTAACCACGGGGGATTATTTATATATAAATCCTGTCAGACTGC

TTCTTTCTGTAGTTGGTGAAGTGTGAGTTCTACTGTTAACGGAGGTAGAGATTTTAGAACATACCAGTAGACAGACTGTGGGACACTCA

ATTAAATGGCAAAACATCCACA
```
